# Personality traits in bipolar disorder and influence on outcome

**DOI:** 10.1186/s12888-017-1332-0

**Published:** 2017-05-03

**Authors:** Timea Sparding, Erik Pålsson, Erik Joas, Stefan Hansen, Mikael Landén

**Affiliations:** 1 Institute of Neuroscience and Physiology, Department of Psychiatry and Neurochemistry, the Sahlgrenska Academy, University of Gothenburg, Gothenburg, Sweden; 2 Department of Medical Epidemiology and Biostatistics, Karolinska Institutet, Stockholm, Sweden; 3 Department of Psychology, University of Gothenburg, Blå Stråket 15, floor 3, SE-413 45 Gothenburg, Sweden; 4 Sahlgrenska University Hospital, Blå Stråket 15, floor 3, SE-413 45 Gothenburg, Sweden

**Keywords:** Personality, Bipolar disorder, SSP, Bipolar disorder type I and II, Neuroticism

## Abstract

**Background:**

The aim was to investigate the personality profile of bipolar disorder I and II, and healthy controls, and to study whether personality influences the course of bipolar disorder.

**Methods:**

One hundred ten patients with bipolar disorder I, 85 patients with bipolar disorder II, and 86 healthy individuals had their personality profile assessed using the Swedish universities Scales of Personality (SSP), an instrument developed to explore personality-related vulnerabilities and correlates of psychiatric disorders. Patients were followed prospectively for 2 years. To assess the impact of Neuroticism, Aggressiveness, and Disinhibition on illness course, we performed logistic regressions with the outcome variables mood episodes (depressive, hypo/manic, mixed), suicide attempts, violence, and the number of sick leave days.

**Results:**

Bipolar disorder I and II demonstrated higher global measures of Neuroticism, Aggressiveness, and Disinhibition as compared with healthy controls. A third of the patients scored ≥1 SD above the population-based normative mean on the global neuroticism measure. The two subtypes of bipolar disorder were, however, undistinguishable on all of the personality traits. In the unadjusted model, higher neuroticism at baseline predicted future depressive episodes and suicide attempts/violent behavior, but this association disappeared when adjusting for baseline depressive symptoms as assessed with MADRS.

**Conclusions:**

A significant minority of the patients scored ≥1 SD above the population mean on the global measures of Neuroticism, Aggressiveness and Disinhibition; scores this high are usually evident clinically. Yet, the personality profile does not seem to have prognostic value over a 2-year period.

## Background

Bipolar disorder is distinguished by episodic and extreme shifts in mood and behavior [1]. Kraepelin (1921) proposed based on observations of almost a thousand patients that ‘there are certain temperaments which may be regarded as rudiments of manic-depressive insanity’ and which form “[…] the point of departure for a morbid process” (p. 278) [2]. The most common temperaments in manic-depressive illness were according to Kraepelin the moody ‘depressive’ temperament, the impulsive ‘manic’ temperament, and the hot-tempered ‘irritable’ temperament. Since this early observation, a number of studies have investigated personality in bipolar disorder but with inconsistent findings (e.g., [3–6]).

A large study by Barnett and colleagues comprising two independent samples of patients with bipolar disorder surveyed personality during euthymia in terms of the NEO Five-Factor model [7]. Results showed that the average bipolar disorder patient scored about one standard deviation above the adult US population mean (norm data) on Neuroticism, thus replicating some of the results from two smaller studies [6, 8]. Bipolar disorder patients also scored close to one standard deviation below the population mean on the Conscientiousness scale, which is indicative of increased impulsiveness. This paralleled a study by Muthadie et al., which found that patients with bipolar disorder type I scored substantially higher than controls on more specific measures of impulsivity [9]. Finally, Barnett et al. reported markedly lower scores on Agreeableness-and Extraversion scales, which suggests an increased tendency towards social aggression and irritability. Jylhä et al. also reported lower-than-normal levels of extraversion in patients with bipolar disorder [6].

Assessing personality could be clinically important because, according to Kraepelin [2], the temperament type might forecast the nature and course of the illness. He noted for example that depressive episodes outnumbered manic episodes in patients of the ‘depressive’ disposition, whereas the opposite was true in patients of the ‘manic’ disposition. This view has to our knowledge, however, been tested in one study only: Barnett et al. [7] investigated the associations between personality scores obtained during euthymia and prospective illness course. They found that elevated Neuroticism along with decreased Extraversion predicted a depression-prone course of the illness, whereas no stable predictor of a manic-prone course was detected.

There are currently two established subtypes of bipolar disorder, type I and type II. Whereas the subtypes are similar with respect to illness severity and share a number of clinical features [10], type II is distinguished from type I by the absence of full-blown manic episodes [1]. Studies comparing personality between these subtypes are scarce. Patients with type II have been reported to score considerably higher on neuroticism-related scales than patients with type I [11, 12]. Another study using Temperament and Character Inventory (TCI) [13] showed that type I and II were similar with respect to the higher dimensions of temperament and character (e.g., novelty seeking, self-transcendence), whereas subjects with type II differed on lower dimensions and were somewhat more impulsive, fatigable, and less resourceful [4]. The latter contrasts, however, with findings from a study in which patients were assessed during a major depressive episode, where type I featured higher scores on impulsivity and aggression-but lower hostility scores-than type II [14]. Finally, three studies found no differences in personality between the two subtypes [6, 15, 16]. Hence, it is as yet uncertain whether personality traits differ between the type I and II subtypes of bipolar disorder.

This study aimed to characterize personality in patients with bipolar disorder, and to assess whether the two subtypes of bipolar disorder differ. We also investigated the association between personality traits and illness course. To these ends, we administered the Swedish universities Scales of Personality (SSP) to mood stabilized bipolar disorder patients and healthy controls. SSP is an instrument explicitly developed to explore personality-related vulnerabilities and correlates of psychiatric disorders [17, 18]. The three personality factors tapped by the SSP - Neuroticism, Disinhibition, and Aggressiveness - correspond broadly to the predisposing temperaments hypothesized by Kraepelin. We used outcome data from the 2-year follow-up.

## Methods

### Participants

Data were collected within the framework of St. Göran Bipolar Project, a longitudinal prospective study that has been described in detail previously [19, 20]. In brief, patients were examined at the outpatient clinic Affective Center at Northern Stockholm Psychiatry in Sweden, which at the time served an urban catchment area with a total population of 316,400 persons over 18 years of age. Virtually all new bipolar patients within the specified catchment area were referred for evaluation to this outpatient unit during the recruitment period. Consecutive new outpatients and continuing patients at the unit were invited to participate provided that they were diagnosed with bipolar disorder. The inclusion criteria for the St. Göran Bipolar Project were >17 years of age, fulfilling the DSM-IV criteria for bipolar disorder type I, type II, NOS, or schizoaffective disorder, bipolar type. Exclusion criteria were inability to complete the standard clinical assessment or incapability of providing informed consent. In the present study, patients with a diagnosis of bipolar disorder type I (*n* = 110) or bipolar disorder type II (*n* = 85) who had completed the SSP were selected for inclusion.

Diagnoses were established using a Swedish version of the Affective Disorder Evaluation (ADE), which is a semi-structured interview developed by the Systematic Treatment Enhancement Program of Bipolar Disorder [21]. The ADE guides the interviewer through a systematic assessment of the patient’s current mental state, psychiatric history, and affective diagnosis according to DSM-IV criteria as per the Structured Clinical Interview for DSM-IV (SCID). In addition, The Mini International Neuropsychiatric Interview (M.I.N.I.) was used to screen for co-morbid psychiatric diagnoses [22]. The ADE and M.I.N.I. interviews were conducted by board-certified psychiatrists working at the Affective Center, or residents in psychiatry completing their training at this unit. To minimize risk of inter-rater bias, a best-estimate diagnostic decision was made based on all information available at admission by a consensus panel of experienced board certified psychiatrists specialized in bipolar disorder. All available sources of information, encompassing patient interview, case records, and-if available-interview with next of kin were utilized in the diagnostic assessment. The ADE also captures age at first symptom, the number of affective episodes, marital-and job status. Overall psychological, social, and occupational functioning was assessed with Global Assessment of Functioning (GAF) [23]. To evaluate depressive and manic symptom severity at the time of the assessment of personality, the Montgomery Åsberg Depression Rating Scale (MADRS) [24] and the Young Ziegler Mania Rating Scale (YMRS) [25] were used. For ethical reasons, patients continued to take their prescribed medications at the time of assessment.

After enrollment, patients underwent annual clinical assessments. The current study employed data from the 2-year follow-up. We used cumulative rates of the number of depressive episodes, of hypomanic/manic episodes, or of a suicide attempt/use of violence to create three binary variables (presence/absence). We also used the total number of sick leave days for the same 2-year period as a measure of absence from work because of the illness.

Details about the recruitment of healthy controls have been described previously [26]. In brief, Statistics Sweden matched each patient on sex and age (+/- 1 month) with seven population-based controls who were living in the same geographical area as patients. These controls were contacted by regular mail and invited to participate; 14% responded and agreed to participate. They were first subjected to a preliminary telephone screening to exclude severe mental health issues, neurological problems, and substance abuse. All eligible controls were then thoroughly examined in person by a psychiatrist using the M.I.N.I. [22] and selected parts of the ADE to exclude psychiatric disorders.

The project was approved by Stockholm Regional Ethical Review Board and a written informed consent was obtained from all participants.

### Swedish universities Scales of Personality, SSP

Personality was assessed using the Swedish universities Scales of Personality (SSP), which is a revised version of Karolinska Scales of Personality [17]. It was initially constructed as a tool to find biological correlates of personality traits predisposing for psychiatric disorder. SSP has been evaluated in several large samples comprising both healthy volunteers and psychiatric patients in Sweden. The correlation of the SSP factors with basic personality dimensions has been confirmed by the correlations with the NEO-PI-R scales in an Estonian study [18].

The SSP is a self-report questionnaire consisting of 91 items, grouped into 13 scales, each based on the response to seven items. The response format is 1 (does not apply at all) to 4 (applies completely). Factor analytic work have shown that the scales are conveniently summarized by three overarching factors: Neuroticism (Lack of assertiveness, Mistrust, Somatic trait anxiety, Psychic trait anxiety, Stress susceptibility and Embitterment), Aggressiveness (Social desirability, Physical trait aggression, Verbal trait aggression and Trait irritability), and Disinhibition (Detachment, Adventure seeking and Impulsiveness) [17].

### Statistical analyses

To investigate differences in personality profiles between patients with bipolar disorder I and II, and healthy controls, we used age-adjusted T-scores-scaled to have a mean of 50 and a standard deviation (SD) of 10 - derived from a large Swedish sample [17]. To investigate overall group differences (independent variable) in Neuroticism-, Aggressiveness- and Disinhibition-related scales (dependent variables), we performed multivariate one-way between-groups analyses of variance (MANCOVA). Statistical diagnostics indicated that Pillai’s Trace was the most appropriate test statistic for evaluating the analysis of Neuroticism-related scales; for the remaining scales, Wilks’ lambda is presented. Initial regression analyses indicated that MADRS (but not YMRS) scores influenced SSP scores significantly (Neuroticism: ß = 0.52; Aggressiveness: ß = 0.24; Disinhibition: ß = 0.22). Therefore, participants’ MADRS scores were entered as covariates in the MANCOVAs. When significant results are obtained with this multivariate test of significance, further information on the relation of each dependent variable can be investigated. Follow up analyses give information on whether there is a statistically significant difference between the adjusted means, in order to decide where the differences lie. The importance of the impact of groups (BP I, BP II, and HC) on personality measured with SSP can be evaluated using effect size. We used Partial Eta Squared (Partial Ƞ^2^), which represents the proportion of explained variance in the dependent variable. Values can range between 0 and 1 and according to guidelines proposed by Cohen (1988), 0.01 is regarded as a small, 0.06 as a medium, and 0.14 as a large effect size.

The impact of Neuroticism, Aggressiveness, and Disinhibition on illness course was assessed with logistic regression analysis. The patients were followed up annually from baseline assessment of personality, at which mood symptoms were measured with MADRS and YMRS. We used data from the 2-year follow-up as dependent variables (presence/absence of a depressive episode, of a hypomanic/manic episode, or of a suicide attempt/use of violence, as well as the number of sick leave days). We used cumulative rates of the total number of sick leave days at the 2-year follow-up as a measure for work ability. The number of sick leave days was not normally distributed. We therefore formed two groups in accordance with Swedish Statistics practice to identify long-term sickness absence: (1) < 120 sick leave days (*n* = 62), and (2) ≥120 sick leave days (*n* = 79) during the 2-year follow-up.

We repeated the logistic regressions with MADRS scores added. The unadjusted odds ratio (OR) was compared with the MADRS-adjusted OR.

## Results

The patient groups differed from healthy controls with regard to MADRS scores at baseline. Patients with bipolar disorder I and II were similar in all aspects of illness course at the follow-up assessment after 2 years (see Table 1).

**Table 1:** Means (SD) for background- and patient variables for patients with bipolar disorder I (BD I), patients with bipolar disorder II (BD II), and healthy controls (HC)

	BD I (*n* = 110)	BD II (*n* = 85)	HC (*n* = 86)
Sex (male/female)	40/70	28/57	38/48
Age^a^	38(13)	39(13)	38(14)
MADRS^a^*	5(5)	9(8)	1(2)
YMRS^a^	1(2)	1(2)	0.45(1)
GAF function^a^	68(10)	67(10)	
GAF symptom^a^	68(11)	66(10)	
Age at disorder onset^a^	20(10)	19(12)	
Presence of hypomania/mania (no/yes)^b^	41/38	39/27	
Presence of depression (no/yes)^b^	32/47	19/47	
Presence of violence/suicide attempt (no/yes)^b^	71/7	59/6	
No of sick leave days^b^	282	298	

^a^Baseline, ^b^Follow-up assessment 2 years after the assessment with SSP at baseline. Data were not available for all participants. *Univariate ANOVA conducted on MADRS scores revealed significant group differences; (*F* (2, 242) = 36.63, *p* < 0.001, partial *η* ^*2*^ *= 0.229.* Post hoc analyses (Games Howell) showed that the three groups differed with regard to depressive symptoms, BD II > BD I > HC

### Group differences in SSP personality scores

**Fig. 1:**
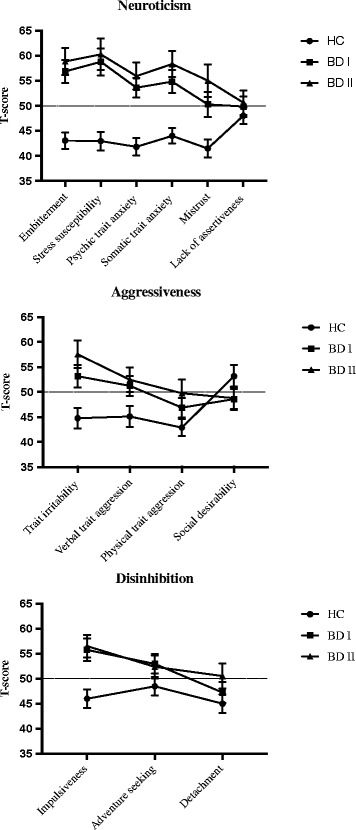
Mean T-scores with 95% CI’s on SSP-derived Neuroticism-, Aggressiveness- and Disinhibition-related subscales in patients with bipolar disorder (BD) I and II, and in healthy controls (HC). The ticked line shows the normative mean for a large Swedish sample (T-score = 50)

Bipolar disorder patients scored higher than healthy controls on most of the SSP scales (Fig. 1). As to the Neuroticism-related scales, MANCOVA (with MADRS scores as covariate) signaled a significant omnibus group difference (F(2, 246) = 7.25, *p* < 0.001, Pillai’s trace = 0.31, *η* ^*2*^ = 0.15).

Subsequent one-way follow-up ANCOVAs and post hoc Scheffé tests of the constituent scales revealed that patients with bipolar I and II disorder scored significantly higher than healthy controls on all of the Neuroticism-related scales except Lack of assertiveness (Table 2). A third of the patients scored ≥1 SD above the population-based normative mean on the global neuroticism measure. The between-groups effect sizes were particularly large with respect to Psychic trait anxiety, Stress susceptibility, and Embitterment (*η* ^*2*^ ~ 0.2). No differences were found between the two bipolar types.

**Table 2:** Results of ANCOVAs on the SSP scales in patients diagnosed with bipolar disorder I (BD I), patients with bipolar disorder II (BD II), and healthy controls (HC)

	*F(2,(df))*	p	*Partial Ƞ* ^*2*^	Pairwise Comparisons^*a*^
*Neuroticism*				
Embitterment	30.35 (247)	0.000	0.20	HC < BD I, BD II
Stress susceptibility	26.7 (247)	0.000	0.18	HC < BD I, BD II
Somatic trait anxiety	17.54 (247)	0.000	0.13	HC < BD I, BD II
Psychic trait anxiety	25.19 (247)	0.000	0.20	HC < BD I, BD II
Mistrust	7.94 (247)	0.000	0.06	HC < BD I, BD II
Lack of assertiveness	0.90 (247)	0.410	0.01	-
Aggressiveness				
Trait irritability	14.18 (248)	0.000	0.10	HC < BD I, BD II
Verbal trait aggression	6.65 (248)	0.002	0.05	HC < BD I, BD II
Physical trait aggression	3.45 (248)	0.033	0.03	HC < BD I, BD II
Social desirability	2.49 (248)	0.085	0.02	HC > BD I
Disinhibition				
Impulsiveness	19.66 (248)	0.000	0.14	HC < BD I, BD II
Adventure seeking	5.12 (248)	0.007	0.04	HC < BD I, BD II
Detachment	1.56 (248)	0.211	0.01	-

^a^ No adjustment of alpha levels. The mean difference is significant at the .05 level

A MANCOVA (with MADRS scores as covariate) of the Aggressiveness-related scales similarly revealed an overall group difference (F(2, 245) = 4.08, *p* < 0.001, Wilks’ lambda = 0.89, *η* ^*2*^ = 0.06; Fig. 1 and Table 2). Follow-up analyses on constituent scales showed that patients with bipolar I and II disorder scored higher than healthy controls on Trait irritability-, Physical trait aggression-, and Verbal trait aggression scales, with the largest difference in Trait irritability (*η* ^*2*^ = 0.10). With regard to Social desirability, only the bipolar I patients differed significantly from controls. Approximately 20% of the patients scored ≥1 SD above the population mean on the global Aggressiveness measure.

The MANCOVA (with MADRS scores as covariate) of the scales measuring aspects of Disinhibition was significant (F(2, 247) = 6.68, *p* < 0.001, Wilks’ lambda = 0.85, *η* ^*2*^ = 0.08; Fig. 1 and Table 2). Patients with bipolar I and II disorder scored higher than healthy controls on all of the scales except Detachment. The Impulsiveness scale had the largest effect size (*η* ^*2*^ = 0.14) in this trait. About 25% of the patients scored ≥1 SD above the population mean on the global Disinhibition measure.

### SSP configurations

**Fig. 2:**
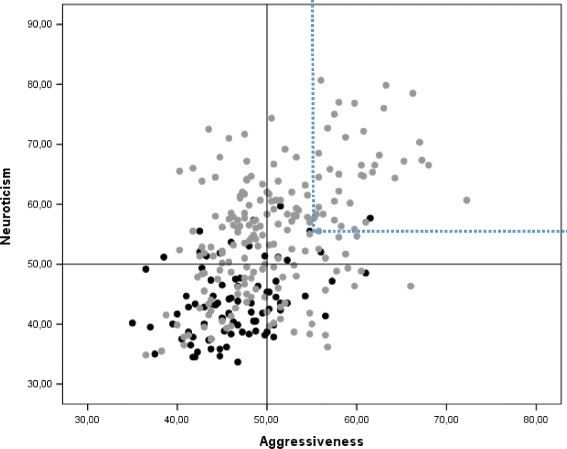
Configurational representation of SSP-derived Neuroticism- and Aggressiveness-related scales (overall mean of all constituent subscales) in patients with bipolar disorder I and II (*grey*) and healthy controls (*black*). The upper right quadrant shows patients that combine high scores (>55 T-scores) on both scales (19.5%)

**Fig. 3:**
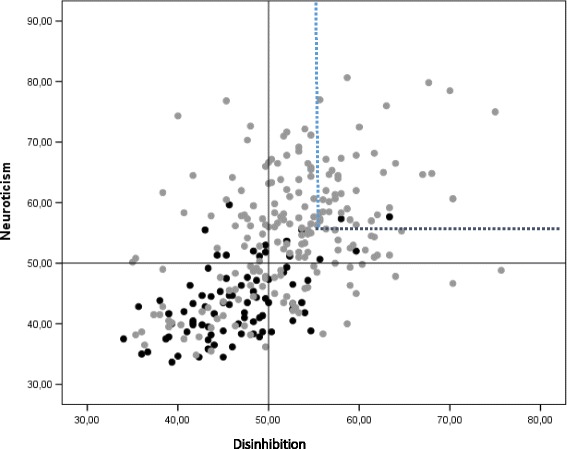
Configurational representation of SSP-derived Neuroticism- and Disinhibition-related scales (overall mean of all constituent subscales) in patients with bipolar disorder I and II (*grey*) and healthy controls (*black*). The upper right quadrant shows patients that combine high scores (>55 T-scores) on both scales (22.1%)

**Fig. 4:**
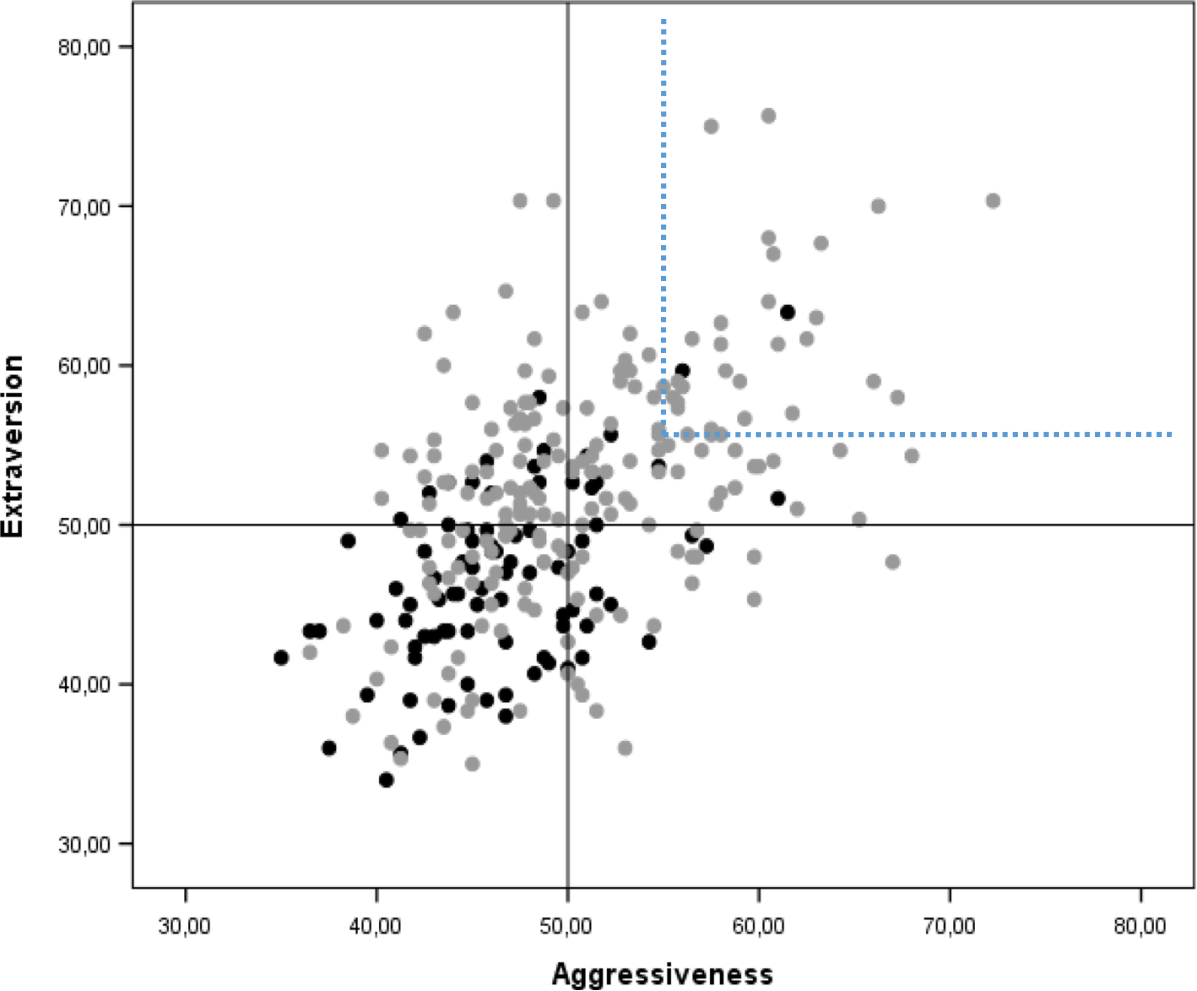
Configurational representation of SSP-derived Disinhibition- and Aggressiveness-related scales (overall mean of all constituent subscales) in patients with bipolar disorder I and II (*grey*) and healthy controls (*black*). The upper right quadrant shows patients that combine high scores (>55 T-scores) on both scales (14.9%)

Figures 2, 3 and 4 show that many patients combined high scores on one of the scales with high scores on the others. For instance, 19.5% scored ≥55 (i.e., >0.5 SD above the population mean) on Neuroticism and Aggressiveness, 22.1% on Neuroticism and Disinhibition, and 14.9% on Aggressiveness and Disinhibition. The corresponding numbers in the control groups were 1.1, 2.3 and 1.1%, respectively. Group differences were significant (all *p*-values <0.01) by the Fisher exact test (data not shown).

### Association between personality and illness course

To assess the impact of Neuroticism, Aggressiveness, and Disinhibition on illness course, we first performed four direct logistic regressions with the dependent variables collected over the 2-year follow-up: presence/absence of any depressive episode, manic/hypomanic episode, or suicide attempt/use of violence and more or less than 120 sick leave days. We repeated the regressions with the addition of MADRS scores. None of the three personality factors was significantly associated with illness course after adjustment for baseline MADRS score (see Table 3).

**Table 3:** Association between SSP personality factors and illness course in participants with bipolar disorder. Odds ratios (OR) with 95% CI intervals are presented

	*Personality factors*	*OR (95% CI)*	*OR (95% CI) adjusted for MADRS*
*any depressive episode*	Neuroticism	1.052(1.007–1.099)^a^	1.036(0.984–1.091)
	Aggressiveness	1.066(0.95–1.14)	1.058(0.981–1.141)
	Disinhibition	0.985(0.929–1.044)	0.986(0.927–1.048)
*anymanic/hypomanic episode*	Neuroticism	1.033(0.993–1.074)	1.012(0.967–1.060)
	Aggressiveness	1.026(0.966–1.089)	1.019(0.955–1.088)
	Disinhibition	0.980(0.929–1.034)	0.987(0.934–1.087)
*any attempt/use of violence*	Neuroticism	1.088(1.016–1.166)^a^	1.039(0.960–1.125)
	Aggressiveness	0.977(0.879–1.087)	0.974(0.869–1.092)
	Disinhibition	0.928(0.844–1.020)	0.924(0.833–1.026)
*sick leave days-more or less than 120 days*	Neuroticism	1.072(1.031–1.116)^a^	1.040(0.994–1.088)
	Aggressiveness	1.019(0.966–1.075)	0.974(0.915–1.037)
	Disinhibition	1.052(1.002–1.104)^a^	1.037(0.986–1.091)

^a^ significant at the .05 level

## Discussion

We examined personality in persons with bipolar disorder type I and II as well as controls, whereafter we followed the patients for 2 years. The main findings were that whilst bipolar disorder patients scored significantly higher than controls on Neuroticism, Extraversion, and Disinhibition, there were no discernible differences between bipolar I and II disorder. In the unadjusted model, higher neuroticism at baseline predicted future depressive episodes and suicide attempts/violent behavior, but this association disappeared when adjusting for baseline depressive symptoms as assessed with MADRS. Personality scores did not differ between patients with high rates of sick leave and those that were able to work.

Patients with bipolar disorder scored higher than controls on most of the neuroticism-related scales. The effect sizes were large with respect to Psychic trait anxiety, Stress susceptibility, and Embitterment. Notably, about one third of patients scored ≥1 SD above the population mean on the global Neuroticism measure; scores this high are evident clinically [27]. The finding that bipolar disorder patients score high on neuroticism concurs with earlier studies [3, 7, 8]. High neuroticism is, however, not specific to bipolar disorder but has been associated with many mental and somatic illnesses [28].

Aggressiveness and Disinhibition were also significantly higher in bipolar disorder patients than controls. Approximately 20% of bipolar patients scored ≥1 SD above the population mean on the global Aggressiveness measure. The high scores on Aggression may correspond to lower scores on Agreeableness and Extraversion that has been found previously in bipolar disorder [7].

Around 25% scored ≥1 SD above the population mean on the global Disinhibition measure. The largest effect sizes were seen for Trait irritability and Impulsiveness. The impulsiveness construct is, however, multi-faceted. Drilling deeper into this construct, Muthadie et al., [9] found that euthymic bipolar I patients were more likely to act on the spur of the moment when experiencing strong emotion. This is in line with a study by Dervic and collegues who found high trait-impulsivity in depressed bipolar patients [14].

Many patients in this study combined high neuroticism scores with high scores on aggressiveness or disinhibition. For example, a fifth of the patients scored >0.5 SD above the population mean on both neuroticism and aggressiveness. This configuration corresponds to a personality characterized by defensive aggression and explosive hostility in response to imagined or real threats, as reviewed by Albert and colleagues [29]. In the same vein, more than a fifth of bipolar disorder patiens combined high scores (>0.5 SD above the population mean) on Neuroticism and Impulsiveness. This pattern of enhanced sensitivities to both punishment and reward [30] would suggest a personality characterized by continual and pervasive approach-avoidance conflicts.

Mood swings and impulsivity are essential features of borderline personality disorder [31]. Borderline personality disorder and bipolar disorder might co-occur and it has been argued that the both illnesses exist on a spectrum, even though this is matter of debate [31]. Considering the significant overlap that several personality system models (e.g., the five factor model) show with personality disorder [32], one would thus expect some differences related to bipolar subtype in the SSP subscales. However, we found no significant differences between the two subtypes of bipolar disorder on any of the 13 SSP subscales. These results are consistent with those of Parker et al. [33] and Fletcher et al. [15] who measured lower-order temperament and personality constructs with Temperament and Personality Questionnaire. By contrast, Akiskal et al. reported that the bipolar subtypes differed on measures capturing “mood lability, assertiveness and brooding”, where the bipolar I subtype scored closer to the comparison group [11].

The interaction between personality and affective disorder is complex with many unresolved issues. As hypothesized by Kraepelin [2] and other pioneers in psychiatry, the deviant personality profile of the average bipolar patient may constitute a premorbid vulnerability for the illness. But there are several other possibilities. First, personality and affective disorder may be independent of each other. Some studies report in fact surprisingly small, albeit statistically significant, differences in personality between euthymic bipolar patients and healthy controls (e.g., [4]). In the present study, ~ 60% of the bipolar patients had unremarkable SSP profiles, with SSP scores falling within ±1SD of the mean (i.e., T-scores between 40 and 60).

Second, personality may modify/complicate the illness [34]. Barnett et al. [7] found for example Neuroticism to be a predictor of lifetime depression. In the present study, we addressed this issue by analysing whether baseline SSP global traits were associated with illness course in terms of depressive and manic/hypomanic episodes, as well as suicide attempts/use of violence and number of sick leave days during a 2-year follow-up period. Controlling for baseline MADRS score, however, we found no associations between the SSP global traits and illness course. Nor did we find any significant differences in personality between the two sick leave groups.

Third, a specific personality profile during euthymia might be a milder, subclinical manifestation of bipolar disorder, as it is assumed that personality and affective episodes express the same genetic endowment [34]. Our study lends some support for this notion by suggesting that bipolar disorder has a unique personality profile being associated with palpable and significant increases in all three of Neuroticism, Aggressiveness, and Disinhibition. Even though high Neuroticism is broadly related to psychopathology [35], this triad is not shared with premenstrual dysphoric disorder [36], obese eating behavior [37], irritable bowel syndrome and social anxiety [27], major depression [38], panic disorder [39], or schizophrenia [40].

Fourth, the illness may alter personality [34]. Longitudinal repeated measures are necessary to address this. We note, however, that SSP profiles seem resistant to life-changing aversive/traumatic events. For example, the SSP scores of people having suffered severe burn trauma fall within normal limits in the long term [41].

Finally we note that the recently identified General Psychopathology Factor [42] is associated with high neuroticism, poorer impulse control, and heightened aggressiveness, i.e., the very personality profile of bipolar disorder identified in the current study. Thus, this profile may express a general tendency to experience persistent and common psychopathologies.

Kraepelin [2] suggested that temperament type might predict the course of the illness.

Reliable predictors, distinguishing between malignant and benign forms of bipolar disorder, would be of great value in mental health care. We found that neuroticism predicted depressive episodes as well as suicidal/violent behavior. This finding accords Kreapelin’s hypothesis and is in line with recent findings by Barnett and coworkers [7]. Importantly though, the association between neuroticism and outcome disappeared when we adjusted for baseline ratings of depressive symptoms using MADRS. It could, however, be argued that subsyndromal depressive symptoms tap in to the neuroticism construct or are an intermediary step in the causal pathway from neuroticism to the outcome of depressive mood episodes. In that case, baseline depressive symptoms would not meet the necessary condition for confounding and the association between neuroticism and outcome should not be adjusted for baseline depressive symptoms. At any rate, our findings suggest that personality assessment at baseline does not seem to have an added value beyond initial depressive symptoms in the prediction of illness course.

### Strengths and limitations

The strengths of this study include a meticulous clinical assessment of patients and healthy controls. We controlled for the confounding effects of baseline depressive symptomatology, which several studies in the field failed to do. This is important because the response to self-report questionnaires may be mood sensitive. Finally, we used a prospective design to study if personality traits could predict the progress of the illness. There are also some limitations to consider. Given that “[t]he human mind operates largely out of view” [43] and that people’s self-knowledge is limited to conscious construals and self-theories [44], our use of a self-report inventory resting on introspection may be questioned. The SSP is also only one of several personality scales and may not capture all traits relevant to bipolar disorder and illness course. Moreover, whereas personality traits are stable over time, symtoms change markedly within the same individual. Personality may thus be associated with vulnerability for the disorder and affecting coping skills, rather than causing symtoms associated with the disorder. Finally, the association between personality and rare events, e.g., suicide attempts might not be captured in this study due to limited power.

## Conclusions

As a group, bipolar disorder patients scored higher than controls on the personality traits Neuroticism, Extraversion, and Disinhibition. Importantly, however, there were no discernible differences between bipolar I and II disorder. The personality scores neither predict occupational functioning, nor other important clinical outcomes in a 2-year follow-up.

## Abbreviations

ADE: Affective Disorder Evaluation
BP I: Bipolar I disorder
BP II: Bipolar II disorder
GAF: Global Assessment of Functioning
HC: healthy controls
M.I.N.I.: The Mini International Neuropsychiatric Interview
MADRS: Montgomery Åsberg Depression Rating Scale
MANCOVA: Multivariate analysis of covariance
OR: odds ratio
Partial Ƞ^2^: Partial Eta Squared
SCID: Structured Clinical Interview for DSM-IV
SD: standard deviation
SSP: Swedish universities Scales of Personality
TCI: Temperament and Character Inventory
YMRS: Young Ziegler Mania Rating Scale
